# Periostin and KIM-1 as Fibrosis-Related Markers Associated with CKD Stage in Children

**DOI:** 10.3390/ijms27083640

**Published:** 2026-04-19

**Authors:** Agnieszka Pukajło-Marczyk, Anna Medyńska, Anna Jakubowska, Maciej Wuczyński, Danuta Zwolińska, Katarzyna Kiliś-Pstrusińska

**Affiliations:** 1 Clinical Department of Paediatric Nephrology, Wroclaw Medical University, ul. Borowska 213, 50-556 Wroclaw, Poland; anna.medynska@umw.edu.pl (A.M.); anna.jakubowska@umw.edu.pl (A.J.); danuta.zwolinska@umw.edu.pl (D.Z.); katarzyna.kilis-pstrusinska@umw.edu.pl (K.K.-P.); 2 Statistical Analysis Centre, Wroclaw Medical University, ul. K. Marcinkowskiego 2–6, 50-368 Wroclaw, Poland; maciej.wuczynski@umw.edu.pl

**Keywords:** chronic kidney disease, fractional excretion, kidney injury molecule-1, Pediatrics, periostin

## Abstract

Early diagnosis of chronic kidney disease (CKD) remains a major clinical challenge. Periostin (POST) and kidney injury molecule-1 (KIM-1) have been proposed as biomarkers of tubular injury and fibrosis. This study aimed to evaluate their utility as markers associated with CKD stage and their associations with renal function and proteinuria in children. Twenty-three children with CKD stages I–IV and 23 healthy controls were enrolled. Serum and urinary POST and KIM-1 were measured together with creatinine (CR), cystatin C (CysC), proteinuria, albuminuria, and urinary α1- and β2-microglobulin. Patients were classified as early stage (ES; CKD I–II) or late stage (LS; CKD III–IV). Serum and urinary POST and KIM-1, uPOST/CR, uKIM-1/CR, fractional excretion indices (FePOST, FeKIM-1), and UPCR were higher in CKD patients than in controls. Absolute biomarker concentrations did not differ between ES and LS and were not associated with eGFR, UPCR, UACR, or tubular protein excretion. In contrast, uPOST/CR, uKIM-1/CR, FePOST, and FeKIM-1 increased with CKD stage, were higher in LS than ES, correlated positively with CysC, and inversely with eGFR. FePOST and FeKIM-1 also correlated strongly with tubular protein markers. The FePOST/FeKIM-1 ratio was elevated in ES patients compared with controls and remained stable across CKD stages. Fractional excretion of POST and KIM-1 is associated with CKD stage and reflects ongoing tubular injury in children. The FePOST/FeKIM-1 ratio may represent a sensitive marker of early CKD.

## 1. Introduction

Chronic kidney disease (CKD) is characterized by a progressive and irreversible decline in renal function, ultimately leading to end-stage kidney disease and the need for renal replacement therapy. Renal fibrosis represents a central pathological mechanism driving CKD progression and is associated with excessive accumulation of extracellular matrix proteins within the renal interstitium. Among biomarkers reflecting renal injury, kidney injury molecule-1 (KIM-1) and periostin (POST) have emerged as important indicators of tubular damage and fibrotic remodeling.

POST, initially described as an osteoblast-specific factor, participates in collagen fibrillogenesis through its EMI domain [1]. In healthy kidneys, POST expression is limited to the vascular pole of the glomerulus and Bowman’s capsule, whereas progressive kidney injury is associated with marked upregulation of POST in renal tissue. Experimental studies suggest that POST interacts with the renin–angiotensin–aldosterone system, induces integrin αvβ3 expression, and modulates transforming growth factor-β (TGF-β) signaling, thereby promoting fibroblast activation, collagen synthesis, and tissue remodeling [2]. Inhibition of POST expression reduces renal fibrosis and collagen deposition within the kidney in experimental models [3]. Clinical studies in adults have demonstrated increased POST expression in diabetic nephropathy, chronic allograft nephropathy [4,5]. In lupus nephritis, higher renal POST expression has been associated with lower glomerular filtration rate (GFR) [6]. Elevated urinary POST levels in adult CKD cohort were significantly higher than in controls, and in patients with type 2 diabetes were correlated positively with albuminuria and declining GFR [7].

KIM-1 is a type I transmembrane glycoprotein belonging to the T-cell immunoglobulin mucin family and is minimally expressed in healthy kidneys [8]. Its expression is markedly upregulated in proximal tubular epithelial cells in response to injury [9,10,11]. KIM-1 participates in the phagocytosis of apoptotic cells and modulates the inflammatory and immune response, thus playing a dual role in the immune response, both protective and pathological [12,13]. Urinary KIM-1 is a highly sensitive marker of acute kidney injury and often precedes changes in serum creatinine [14]. Through its role in clearing apoptotic cells, KIM-1 helps to attenuate inflammation and promote tissue repair [15].

In CKD, sustained KIM-1 expression contributes to chronic inflammation and fibrosis, promoting disease progression. Elevated urinary and plasma KIM-1 levels correlate with CKD severity and independently predict disease progression [16,17,18].

Despite growing evidence supporting the role of POST and KIM-1 as markers of renal injury in adults, their significance in pediatric CKD remains poorly investigated.

Given the ongoing renal development and dynamic physiological changes during childhood, studies in pediatric populations are particularly warranted.

Aim of study

The aim of this study was to evaluate the usefulness of POST and KIM-1 as biomarkers associated with CKD stage in pediatric patients. Assessing the relationship between CKD stage and urinary excretion of POST and KIM-1 may provide insight into their potential role in tubular injury and processes related to renal fibrogenesis. Early assessment of these biomarkers may facilitate the identification of children at risk and support earlier specialist follow-up.

The significance of POST in the pediatric CKD population has not yet been investigated, and therefore studies in this age group are warranted.

## 2. Results

Table 1 presents the basic demographic characteristics, kidney function indices, and the evaluation of proteinuria for the CKD and control groups, as well as for subgroups classified according to CKD stage (ES and LS).

Evaluation of serum and urinary concentrations of POST and KIM-1 revealed markedly higher values in children with CKD compared with healthy controls. Similar findings were observed for the urinary ratios uPOST/CR and uKIM-1/CR, as well as for the fractional excretion indices FePOST and FeKIM-1, all of which were significantly elevated in the CKD group (Table 2).

**Figure 1:**
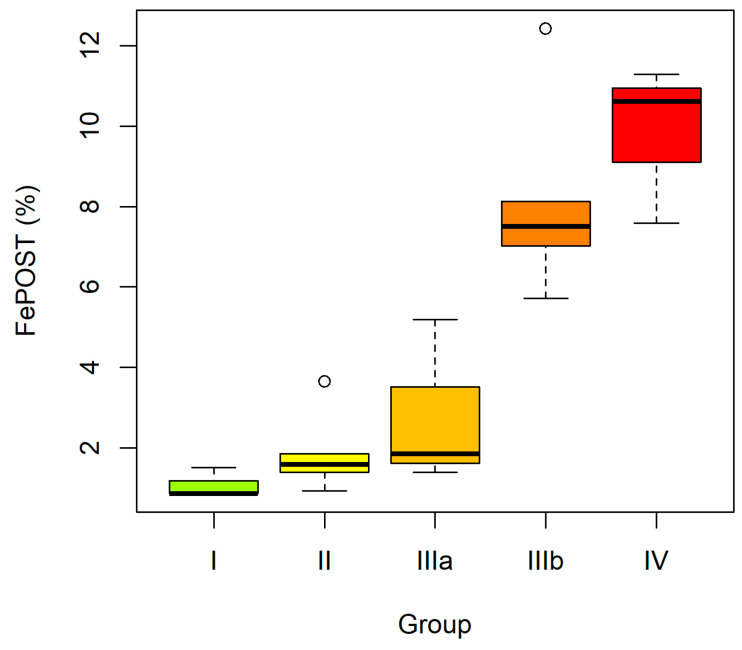
Fractional excretion of periostin (FePOST) in CKD subgroups according to disease. stages: I–II–IIIa–IIIb–IV. Boxes indicate the interquartile range (IQR), the median is shown as a line within the box, whiskers represent the minimum and maximum values, and outliers (values exceeding 1.5× IQR) are shown as open circles.

**Figure 2:**
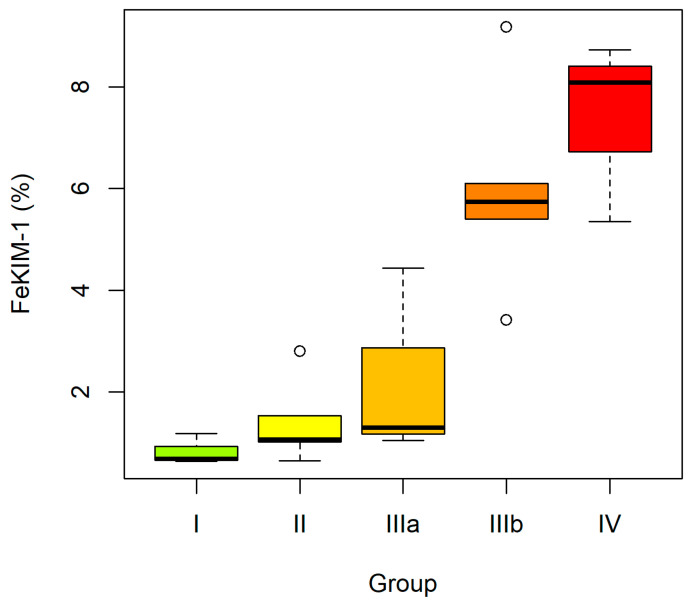
Fractional excretion of KIM-1 (FeKIM-1) in CKD subgroups according to disease. stages: I–II–IIIa–IIIb–IV. Boxplots constructed as in Figure 1.

When sPOST, uPOST, sKIM-1 and uKIM-1 were analyzed in CKD subgroups (ES vs. LS), the absolute concentrations of the studied proteins did not differ significantly between the groups. The trend analysis likewise did not demonstrate significant changes in serum or urinary concentrations of POST and KIM-1 with disease progression. However, the urinary ratios (uPOST/CR and uKIM-1/CR) and the fractional excretion indices (FePOST and FeKIM-1) showed a dynamic increase, with significantly higher values in the LS group compared with the ES group. Moreover, a statistically significant upward trend of these indices was observed, indicating a progressive increase with advancing CKD stage: I–II–IIIa–IIIb–IV (Figure 1 and Figure 2).

**Figure 3:**
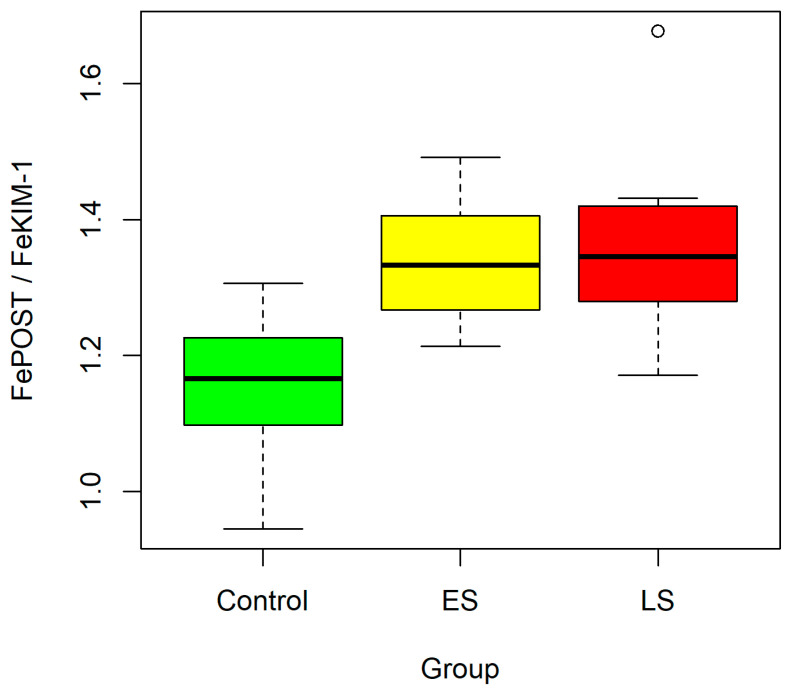
FePOST/FeKIM-1 ratio in the control group compared to the group of children with early (ES) and late stage (LS) of CKD. Boxplots constructed as in Figure 1.

The FePOST/FeKIM-1 ratio was significantly higher in the CKD group compared with the control group. However, no statistically significant differences were observed between the ES and LS subgroups, and no upward trend was demonstrated across CKD stages (Figure 3).

Evaluation of proteinuria, expressed as UPCR, confirmed significantly higher values in the CKD group compared with the control group. No significant differences were observed between the ES and LS subgroups (Table 1). A similar pattern was noted for the UACR, whose values did not differ between CKD subgroups and showed no upward trend with disease progression (Table 3).

**Figure 4:**
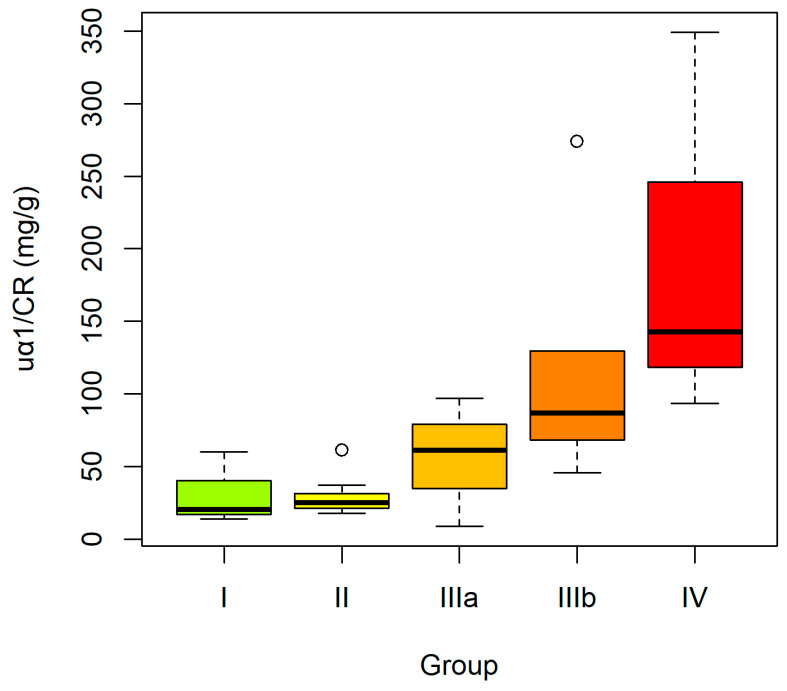
Urinary α1-microglobulin-to-creatinine ratio (uα1/CR) in CKD subgroups according to disease stages: I–II–IIIa–IIIb–IV. Boxplots constructed as in Figure 1.

**Figure 5:**
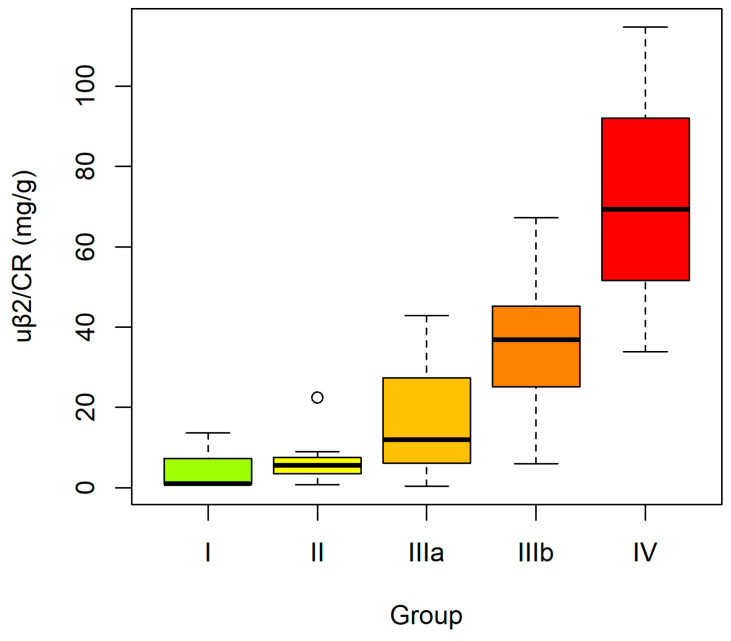
Urinary β2-microglobulin-to-creatinine ratio (uβ2/CR) in CKD subgroups according to disease stages: I–II–IIIa–IIIb–IV. Boxplots constructed as in Figure 1. Boxplots constructed as in Figure 1.

In contrast, the uα1/CR and uβ2/CR demonstrated a clear increase with advancing CKD stage. Both comparative and trend analyses confirmed the statistically significant dynamics of these parameters in relation to disease severity (Figure 4 and Figure 5).

Correlation analysis in the CKD group showed that serum and urinary concentrations of POST and KIM-1 did not correlate with serum creatinine, cystatin C, eGFR based on CR or CysC, nor with UPCR or the excretion of tubular proteins. In contrast, the ratios uPOST/CR and uKIM-1/CR, as well as FePOST and FeKIM-1, showed strong positive correlations with serum CR and CysC, and negative correlations with both eGFR CR and eGFR CysC. None of these indices correlated with UPCR or UACR. However, FePOST and FeKIM-1 were the only indices that exhibited strong positive correlations with the excretion of tubular proteins—uα1/CR and uβ2/CR.

The indices uKIM-1/CR and uPOST/CR were positively correlated with each other and with both FePOST and FeKIM-1. The results of the correlation analysis in the CKD group are presented in Table 4. Similar relationships between these parameters were confirmed in the control group. The FePOST/FeKIM-1 ratio did not show any correlation with the analyzed markers of kidney function or proteinuria, either in the CKD group or in healthy controls (Table 4).

Relationships between the studied biomarkers and CKD stage were additionally evaluated using partial correlation analysis (Table 5). In most cases, the biomarkers remained significantly associated with CKD stage after adjustment for established predictors of kidney function, suggesting that these associations are not solely driven by creanine-related effects. Notably, creatinine-normalized and fractional excretion indices retained their associations with CKD stage after adjustment.

Additionally, ROC curve analysis demonstrated that several predictors evaluated in this study showed excellent discriminatory performance between healthy controls and CKD subgroups. Specifically, creatinine, eGFR, cystatin C (CysC), sPOST, uPOST, sKIM-1, and uKIM-1 achieved an AUC of 1.0 in at least one binary comparison. Detailed ROC results are provided in the Supplementary Materials—Table S1.

## 3. Discussion

The primary aim of this study was to evaluate the prognostic value of periostin (POST) and kidney injury molecule-1 (KIM-1) in children with CKD and to compare them with established indicators of disease progression, including proteinuria. To our knowledge, this is the first study to assess serum and urinary POST in pediatric CKD.

Satirapoj et al. demonstrated increased POST expression in experimental models of renal injury and showed that urinary POST levels were elevated in adult CKD patients regardless of proteinuria status. These findings suggested that urinary POST reflects tubular injury rather than glomerular damage [19]. Our results are consistent with these observations, as uPOST and uPOST/CR were higher in CKD patients than in controls and did not correlate with proteinuria. These findings are therefore in line with previous observations, suggesting that proteinuria does not directly affect urinary periostin levels.

McDonnell et al. reported that plasma and urinary KIM-1 concentrations were associated with CKD progression and adverse outcomes in adults [20]. In a cohort of CKD children, Greenberg at al. identified plasma KIM-1 as one of four biomarkers which may improve risk prediction of the disease progression [18]. Similarly, we observed significantly higher serum and urinary KIM-1 levels in children with CKD. However, absolute POST and KIM-1 concentrations did not differ between early and late disease stages and did not show dynamic changes with disease progression.

In contrast, indices normalized to urinary creatinine (uPOST/CR, uKIM-1/CR) and fractional excretion parameters (FePOST, FeKIM-1) increased progressively with advancing CKD and correlated strongly with cystatin C and eGFR (eGFR CR and eGFR CysC). These findings indicate that normalized and fractional excretion indices better reflect disease severity than absolute biomarker concentrations. These observations were further supported by partial correlation analysis, which demonstrated that the associations between creatinine-normalized and fractional excretion indices and CKD stage remained significant after adjustment for established predictors of kidney function. This suggests that the observed relationships are not solely driven by creatinine-related effects but may reflect biologically relevant alterations in tubular handling of these proteins.

In addition, ROC curve analysis confirmed the strong discriminatory performance of the analyzed biomarkers, with several parameters achieving excellent or even perfect classification between CKD subgroups. Together, these findings strengthen the robustness of the observed associations.

Moreover, uPOST/CR and uKIM-1/CR values gradually increased with the advancement of CKD, but still did not correlate with tubular proteinuria, which usually reflects advanced tubular damage.

Assessment of fractional excretion provides a more pathophysiologically meaningful measure, as it accounts for serum concentrations and reflects the proportion of filtered or locally produced protein excreted in urine. Previous studies have demonstrated the prognostic value of fractional excretion of immunoglobulin G and of high- and low–molecular-weight proteins as potential prognostic markers in FSGS and other renal pathologies [21,22]. In our study, FePOST and FeKIM-1 showed strong positive correlations with tubular proteinuria and no association with glomerular proteinuria, supporting their tubular origin. Although creatinine-normalized and fractional excretion indices are mathematically related to serum and urinary creatinine, the persistence of significant associations with CKD stage after adjustment for kidney function parameters suggests that these findings are not merely driven by mathematical coupling.

The increase in FePOST and FeKIM-1 despite stable serum concentrations may reflect altered tubular handling of these proteins, potentially involving increased local production and/or changes in reabsorption rather than increased filtration. The strong correlation between FePOST and FeKIM-1 may suggest a shared tubular origin and a possible link to CKD-related fibrotic processes; however, these mechanisms require further investigation.

The observed increase in FePOST and FeKIM-1 with declining eGFR highlights their potential utility as markers associated with CKD severity. These findings may reflect ongoing tubular injury and mechanisms potentially related to fibrotic processes. However, given the relatively small sample size and subgroup stratification, the present study should be considered exploratory in nature, and the findings require confirmation in larger pediatric cohorts. Interestingly, the FePOST/FeKIM-1 ratio was elevated in CKD patients compared with controls but remained stable across disease stages, suggesting that coordinated upregulation of both markers may occur early in CKD and persist into more advanced stages of the disease.

## 4. Materials and Methods

The study included 46 children: 23 patients with CKD stages I–IV and 23 healthy controls. In the CKD group, most patients (20/23) had congenital anomalies of the urinary tract, while one had Alport syndrome and two had a history of hemolytic–uremic syndrome. Importantly, none of the patients in our cohort were treated with steroids or other immunosuppressive agents. The CKD group consisted of 10 girls and 13 boys (mean age 154.3 ± 41.1 months) and was divided into early-stage (ES, stages I–II; *n* = 10, mean age 144.00 ± 38.08 months) and late-stage (LS, stages III–IV; *n* = 13, mean age 162.15 ± 43.06 months) subgroups. The control group comprised 23 children (11 girls, 12 boys; mean age 129.8 ± 39.7 months) evaluated for nocturnal enuresis or suspected urinary tract abnormalities that were ultimately excluded. All control subjects had normal renal function, no proteinuria, and no evidence of urinary tract abnormalities.

Fasting blood samples and first-morning urine samples were collected during routine clinical visits. After centrifugation, samples were stored at −70 °C until analysis.

Serum and urinary concentrations of POST and KIM-1 were measured using ELISA kits (Sunlong Biotech System, Sunlong Biotech Co., Ltd., Hangzhou, China; POST: SL2267Hu, KIM-1: SL1031Hu) according to the manufacturer’s instructions. The analytical sensitivity and measurement range were provided by the manufacturer. The intra- and inter-assay coefficients of variation were <10% and <12% for both POST and KIM-1 assays. All samples were analysed in duplicate. Creatinine (CR), cystatin C (CysC), urea, BUN, uric acid, and C-reactive protein (CRP) were measured in serum, while urinary creatinine, total protein, albumin, and tubular proteins (α1- and β2-microglobulin) were assessed using standard laboratory methods.

Estimated glomerular filtration rate (eGFR) was calculated using the revised Schwartz formula (eGFR CR) and a cystatin C–based equation (eGFR CysC) [23,24]. Proteinuria was evaluated using urinary protein-to-creatinine (UPCR), albumin-to-creatinine (UACR), and tubular protein-to-creatinine (uα1/CR, uβ2/CR) ratios.

Urinary excretion of POST and KIM-1 was expressed as urinary concentration-to-creatinine ratios (uPOST/CR, uKIM-1/CR). Due to the wide variability of serum and urine creatinine concentrations depending on the stage of kidney disease, the fractional excretion of these proteins (FePOST and FeKIM-1) was additionally calculated using the standard formula: FeX = [(urinary X × serum creatinine)/(serum X × urinary creatinine)] × 100%.

The FePOST/FeKIM-1 ratio was additionally calculated to assess the variability of this relationship according to the degree CKD progression.

### Statistical Methods

Data are presented as medians and interquartile ranges. Normality was assessed using the Shapiro–Wilk test. If both groups showed non-significant results, indicating normal distribution, the homogeneity of variances was evaluated using Levene’s test. When both assumptions (normality and homoscedasticity) were met, Student’s t-test was used for group comparison. In cases of unequal variances, Welch’s t-test was applied instead. If the data in at least one group deviated from normality, the non-parametric Mann–Whitney U test was used. The same analytical approach was applied to subgroup comparisons within the CKD group. Specifically, ES patients (stages I–II) were compared with LS patients (stages III–IV). Trends across CKD stages were evaluated using the Jonckheere–Terpstra test. Associations were analysed using Spearman’s rank correlation and partial correlation analysis. Clinical utility of biomarkers was assessed using ROC curve analysis. Statistical significance was set at *p* ≤ 0.05. Analyses were performed using R software (version 4.3.3) and its libraries: clinfun (v.1.1.5), ppcor (v.1.1) and pROC (v.1.19.0.1).

## 5. Conclusions

This study demonstrates the potential clinical utility of POST and KIM-1 measurements in pediatric CKD. Unadjusted urinary concentrations of these biomarkers were insufficient to discriminate between early and late disease stages. In contrast, creatinine-normalized indices (uPOST/CR, uKIM-1/CR) and fractional excretion parameters (FePOST, FeKIM-1) were associated with CKD stage, showing a stepwise increase with advancing disease severity.

Among all analyzed indices, FePOST and FeKIM-1 appeared to be the most informative. Despite comparable serum concentrations, their fractional excretion correlated strongly with tubular proteinuria but not with glomerular proteinuria, suggesting increased tubular release of POST and KIM-1 associated with tubular injury and mechanisms potentially related to fibrotic processes.

Furthermore, the FePOST/FeKIM-1 ratio was elevated in early-stage CKD and remained stable across disease stages, suggesting its potential as a sensitive marker of early disease onset. These findings should be interpreted with caution and warrant further validation in larger pediatric cohorts.

## Figures and Tables

**Table 1:** Summarizes the basic demographic data, kidney function parameters, and assessment of proteinuria in the CKD and control groups, including subgroup analysis by CKD stage: I–II (early stage, ES) and III–IV (late stage, LS). Data are presented as median values and interquartile ranges.

**Parameter**	**CKD Group** (*n* = 23)	**Control Group** (*n* = 23)	***p*** **(CKD vs. C)**	**ES Group** (*n* = 10)	**LS Group** (*n* = 13)	***p*** **(ES vs. LS)**
Age (months)	161.00 (135.50–188.00)	137.00 (97.00–155.50)	0.0459	145.00 (115.50–161.75)	169.00 (141.00–197.00)	0.3044
Height (cm)	147.00 (132.50–162.00)	147.00 (131.25–163.25)	0.8356	142.25 (134.50–160.00)	149.00 (130.50–164.00)	0.7305
Body weight (kg)	44.30 (29.90–52.73)	40.20 (28.38–55.98)	0.5616	44.65 (28.85–47.39)	43.20 (33.30–57.45)	0.9735
CRP (mg/L)	0.47 (0.40–1.54)	0.40 (0.40–0.83)	0.7871	0.40 (0.39–0.71)	1.39 (0.40–2.04)	0.2082
CR (mg/dL)	1.56 (1.06–2.38)	0.60 (0.57–0.67)	<0.0001	0.97 (0.86–1.19)	2.12 (1.65–3.27)	0.0005
eGFR CR (ml/min/1.73 m^2^)	52.53 (35.12–77.24)	129.32 (119.60–148.42)	<0.0001	79.09 (75.47–92.78)	36.39 (30.70–50.49)	<0.0001
Urea (mg/dL)	54.00 (42.50–88.00)	23.00 (19.00–27.50)	<0.0001	38.50 (32.75–47.75)	82.00 (61.00–95.00)	<0.0001
BUN	25.20 (19.83–41.07)	10.73 (8.87–12.83)	<0.0001	17.97 (15.28–22.28)	38.27 (28.47–44.33)	<0.0001
Uric acid (mg/dL)	6.60 (5.75–6.90)	4.00 (3.70–4.75)	<0.0001	6.80 (5.85–7.55)	6.20 (5.80–6.70)	0.6024
CysC (mg/L)	2.12 (1.41–2.89)	0.81 (0.74–0.91)	<0.0001	1.41 (1.36–1.74)	2.77 (2.33–3.31)	0.0001
eGFR CysC (ml/min/1.73 m^2^)	35.00 (26.00–51.50)	86.00 (77.50–93.50)	<0.0001	51.50 (42.00–52.75)	27.00 (23.00–32.00)	0.0006
uCR (mg/dL)	40.86 (24.74–59.33)	83.55 (42.17–108.20)	0.0044	46.37 (40.63–74.17)	29.13 (19.73–43.34)	0.0326
uProtein (mg/dL)	0.21 (0.14–0.76)	0.11 (0.05–0.17)	0.0069	0.21 (0.10–0.65)	0.22 (0.15–0.71)	0.7415
UPCR (mg/mg)	0.59 (0.27–1.51)	0.13 (0.11–0.17)	<0.0001	0.35 (0.19–0.90)	0.87 (0.37–3.54)	0.1563

CRP—C-reactive protein; CR—creatinine; eGFR—estimated glomerular filtration rate based on creatinine; BUN—blood urea nitrogen; CysC—cystatin C; eGFR CysC—estimated glomerular filtration rate based on cystatin C; u—urine; UPCR—urine protein/creatinine ratio.

**Table 2:** Comparative analysis of urinary biomarkers POST and KIM-1 between children with CKD and healthy controls, as well as between the early-stage (ES) and late-stage (LS) CKD subgroups. Trend analysis for the studied biomarkers across CKD stages I, II, IIIa, IIIb, and IV was performed using the Jonckheere–Terpstra test. Data are presented as median values and interquartile ranges.

**Parameter**	**CKD Group** (*n* = 23)	**Control Group** (*n* = 23)	***p*** **(CKD vs. C)**	**ES Group** (*n* = 10)	**LS Group** (*n* = 13)	***p*** **(ES vs. LS)**	***p*** **Trend** **(ES + LS)**
sPOST (pg/mL)	170.78 (169.49–173.52)	147.90 (145.01–150.71)	<0.0001	172.21 (168.73–173.58)	170.19 (169.72–171.14)	0.7758	>0.9999
uPOST (pg/mL)	136.54 (132.09–138.75)	108.43 (105.66–111.36)	<0.0001	135.68 (133.67–138.64)	137.12 (132.09–139.12)	0.6942	0.8692
uPOST/CR (pg/mg)	321.34 (267.42–586.03)	127.45 (97.91–261.74)	0.0003	299.05 (174.69–319.85)	476.66 (307.53–671.85)	0.0163	0.0085
FePOST (%)	4.42 (1.48–7.62)	0.56 (0.39–1.10)	<0.0001	1.45 (0.92–1.65)	7.44 (5.59–8.75)	0.0005	<0.0001
sKIM-1 (pg/mL)	118.91 (115.72–121.11)	60.23 (58.38–62.15)	<0.0001	119.52 (118.60–119.83)	117.41 (114.16–122.34)	0.3683	0.8916
uKIM-1 (pg/mL)	70.21 (68.73–72.25)	38.04 (36.80–40.21)	<0.0001	72.11 (69.38–72.98)	69.87 (68.16–71.53)	0.0993	0.181
uKIM-1/CR (pg/mg)	173.99 (131.90–303.00)	45.84 (34.11–93.58)	<0.0001	149.86 (93.01–176.23)	253.10 (157.17–349.68)	0.0219	0.0106
FeKIM-1 (%)	3.11 (1.06–5.71)	0.50 (0.33–0.89)	<0.0001	1.04 (0.67–1.27)	5.54 (4.17–6.59)	0.0005	<0.0001
FePOST/ FeKIM-1	1.35 (1.27–1.42)	1.17 (1.10–1.23)	<0.0001	1.33 1.27–1.38)	1.35 (1.29–1.42)	0.7916	0.8692

sPOST—periostin in serum; uPOST—periostin in urine; sKIM-1—KIM-1 in serum; uKIM-1—KIM-1 in urine; uPOST/CR—urine periostin/creatinine ratio; uKIM-1/CR—urine KIM-1/creatinine ratio; FePOST—fractional excretion of periostin; FeKIM-1—fractional excretion of KIM-1; FePOST/FeKIM-1—FePOST/FeKIM-1 ratio.

**Table 3:** Comparative and trend analysis of microalbuminuria and tubular protein excretion in early-stage (ES) and late-stage (LS) CKD subgroups. Data are presented as median values and interquartile ranges.

**Parameter**	**ES Group** (*n* = 10)	**LS Group** (*n* = 13)	***p*** **(ES vs. LS)**	***p* Trend** **(ES vs. LS)**
UACR (mg/g))	167.80 (111.75–857.27)	251.90 (113.65–1989.60)	0.7513	0.5171
uα1 (mg/L)	12.90 (9.05–15.85)	28.60 (15.40–48.20)	0.0545	0.0254
uβ2 (mg/L)	2.62 (0.76–3.37)	7.58 (3.65–22.70)	0.0217	0.0054
uα1/CR (mg/g)	23.15 (20.59–34.35)	95.34 (66.55–132.93)	0.0022	<0.0001
uβ2/CR (mg/g)	5.48 (1.13–8.21)	38.31 (21.86–50.71)	0.0041	<0.0001

UACR—urine albumin/creatinine ratio, uα1—alfa-1 mikroglobulin in urine; uβ2—beta-2 mikroglobulin in urine, uα1/CR—urine alfa-1 mikroglobulin/creatinine ratio, uβ2/CR—urine beta-2 mikroglobulin/creatinine ratio.

**Table 4:** Correlations between serum and urine biomarker concentrations and indices with kidney function parameters and proteinuria in children with CKD.

Parameter	CR (mg/dL)	eGFR CR (mL/min/1.73 m^2^)	Cys C (mg/L)	eGFR CysC (mL/min/1.73 m^2^)	UPCR (mg/mg)	UACR (mg/g)	uα1/CR (mg/g)	uβ2/CR (mg/g)	uPOST/CR (pg/mg)	uKIM-1/CR (pg/mg)
sPOST (pg/mL)										
uPOST (pg/mL)						r = −0.59 *p* = 0.0096				
sKIM-1 (pg/mL)										
uKIM-1 (pg/mL)										
uPOST/CR (pg/mg)	r = 0.46 *p* = 0.0415	r = −0.55 *p* = 0.0129	r = 0.55 *p* = 0.0138	r = −0.54 *p* = 0.0133						r = 1 *p* < 0.0001
uKIM-1/CR (pg/mg)		r = −0.53 *p* = 0.0167	r = 0.54 *p* = 0.0157	r = −0.5344 *p* = 0.0152					r = 1 *p* < 0.0001	
FePOST (%)	r = 0.83 *p* < 0.0001	r = −0.84 *p* < 0.0001	r = 0.82 *p* < 0.0001	r = −0.81 *p* < 0.0001			r = 0.69 *p* = 0.001	r = 0.61 *p* = 0.005	r = 0.84 *p* < 0.0001	r = 0.83 *p* < 0.0001
FeKIM-1 (%)	r = 0.78 *p* < 0.0001	r = −0.82 *p* < 0.0001	r = 0.83 *p* < 0.0001	r = −0.82 *p* < 0.0001			r = 0.67 *p* = 0.0017	r = 0.57 *p* = 0.0103	r = 0.88 *p* < 0.0001	r = 0.88 *p* < 0.0001
FePOST/ FeKIM										

sPOST—periostin in serum; uPOST—periostin in urine; sKIM-1—KIM-1 in serum; uKIM-1—KIM-1 in urine; uPOST/CR—urine periostin/creatinine ratio; uKIM-1/CR—urine KIM-1/creatinine ratio; FePOST—fractional excretion of periostin; FeKIM-1—fractional excretion of KIM-1; FePOST/FeKIM-1—FePOST/FeKIM-1 ratio; CR—creatinine; eGFR CR—estimated glomerular filtration rate based on creatinine; CysC—cystatin C; eGFR CysC—estimated glomerular filtration rate based on cystatin C, UPCR—urine protein/creatinine ratio, UACR—urine albumin/creatinine ratio, uα1/CR—urine alfa-1 mikroglobulin/creatinine ratio, uβ2/CR—urine beta-2 mikroglobulin/creatinine ratio.

**Table 5:** Partial correlations between serum and urine biomarker concentrations and CKD stage, corrected by kidney function parameters and in children with CKD.

Parameter	CR (mg/dL)	eGFR CR (ml/min/1.73 m^2^)	Cys C (mg/L)	eGFR CysC (ml/min/1.73 m^2^)	urine CR (mg/dL)	uα1/CR (mg/g)	uβ2/CR (mg/g)
uPOST/CR (pg/mg)	r = 0.49 *p* = 0.001	r = 0.36 *p* = 0.019	r = 0.33 *p* = 0.032	r = 0.49 *p* = 0.001	r = 0.43 *p* = 0.004	r = 0.48 *p* = 0.039	r = 0.52 *p* = 0.023
uKIM-1/CR (pg/mg)	r = 0.64 *p* < 0.001	r = 0.40 *p* = 0.008	r = 0.45 *p* = 0.003	r = 0.56 *p* < 0.001	r = 0.65 *p* < 0.001	r = 0.46 *p* = 0.045	r = 0.51 *p* = 0.026
FePOST (%)	r = 0.42 *p* = 0.006	r = 0.48 *p* = 0.001	r = 0.22 *p* = 0.154	r = 0.67 *p* < 0.001	r = 0.78 *p* < 0.001	r = 0.72 *p* < 0.001	r = 0.67 *p* = 0.002
FeKIM-1 (%)	r = 0.40 *p* = 0.008	r = 0.46 *p* = 0.002	r = 0.18 *p* = 0.246	r = 0.64 *p* < 0.001	r = 0.75 *p* < 0.001	r = 0.70 *p* = 0.001	r = 0.65 *p* = 0.003
FePOST/ FeKIM	r = 0.41 *p* = 0.007	r = 0.24 *p* = 0.133	r = 0.46 *p* = 0.002	r = 0.40 *p* = 0.009	r = 0.54 *p* < 0.001	r = 0.21 *p* = 0.391	r = 0.07 *p* = 0.773

uPOST/CR—urine periostin/creatinine ratio; uKIM-1/CR—urine KIM-1/creatinine ratio; FePOST—fractional excretion of periostin; FeKIM-1—fractional excretion of KIM-1; FePOST/FeKIM-1—FePOST/FeKIM-1 ratio; CR—creatinine; eGFR CR—estimated glomerular filtration rate based on creatinine; CysC—cystatin C; eGFR CysC—estimated glomerular filtration rate based on cystatin C, uα1/CR—urine alfa-1 mikroglobulin/creatinine ratio, uβ2/CR—urine beta-2 mikroglobulin/creatinine ratio.

## Data Availability

The original contributions presented in this study are included in the article/Supplementary Material. Further inquiries can be directed to the corresponding author.

## Supplementary Materials

The following supporting information can be downloaded at: https://www.mdpi.com/article/10.3390/ijms27083640/s1.
