# Supplementary material for: Periostin and KIM-1 as Fibrosis-Related Markers Associated with CKD Stage in Children
Source: Int J Mol Sci. 2026 Apr 19;27(8):3640. doi: 10.3390/ijms27083640 (PMC13116152; doi:10.3390/ijms27083640)
Supplement: Supplementary file 1 [file ijms-27-03640-s001.zip › ijms-4222450-supplementary.pdf]

Table S1. Results of ROC analysis measuring prediction power of biomarkers used in the study. AUC indicates Area under ROC curve, Sens and Spec indicates Sensitivity and Specificity, respectivelty, with optimal prediction threshold.

C vs CKD	C vs Early Stage	Early Stage vs Late Stage	Parameter
AUC = 0.67<br>Sens = 0.78<br>Spec = 0.57	AUC = 0.61<br>Sens = 0.30<br>Spec = 0.90	AUC = 0.63<br>Sens = 0.80<br>Spec = 0.54	**Age**<br>(months)
AUC = 0.49<br>Sens = 0.22<br>Spec = 0.87	AUC = 0.51<br>Sens = 0.70<br>Spec = 0.50	AUC = 0.48<br>Sens = 0.60<br>Spec = 0.62	**Height**<br>(cm)
AUC = 0.48<br>Sens = 0.57<br>Spec = 0.57	AUC = 0.48<br>Sens = 0.17<br>Spec = 1.00	AUC = 0.47<br>Sens = 1.00<br>Spec = 0.23	**Body weight**<br>(kg)
AUC = 0.52<br>Sens = 0.74<br>Spec = 0.52	AUC = 0.42<br>Sens = 0.74<br>Spec = 0.40	AUC = 0.66<br>Sens = 0.80<br>Spec = 0.54	**CRP**<br>(mg/l)
**AUC = 1.00**<br>**Sens = 1.00**<br>**Spec = 1.00**	**AUC = 1.00**<br>**Sens = 1.00**<br>**Spec = 1.00**	AUC = 0.94<br>Sens = 0.80<br>Spec = 1.00	**CR**<br>(mg/dl)
**AUC = 1.00**<br>**Sens = 1.00**<br>**Spec = 1.00**	**AUC = 1.00**<br>**Sens = 1.00**<br>**Spec = 1.00**	**AUC = 1.00**<br>**Sens = 1.00**<br>**Spec = 1.00**	**eGFR CR**<br>(ml/min/1,73m^2)
AUC = 0.95<br>Sens = 0.87<br>Spec = 0.96	AUC = 0.88<br>Sens = 0.87<br>Spec = 0.90	AUC = 0.97<br>Sens = 1.00<br>Spec = 0.92	**Urea**<br>(mg/dl)
AUC = 0.95<br>Sens = 0.87<br>Spec = 0.96	AUC = 0.88<br>Sens = 0.87<br>Spec = 0.90	AUC = 0.97<br>Sens = 1.00<br>Spec = 0.92	**BUN**
AUC = 0.87<br>Sens = 0.83<br>Spec = 0.87	AUC = 0.83<br>Sens = 0.87<br>Spec = 0.80	AUC = 0.64<br>Sens = 0.50<br>Spec = 0.92	**Uric acid**<br>(mg/dl)
**AUC = 1.00**<br>**Sens = 1.00**<br>**Spec = 1.00**	**AUC = 1.00**<br>**Sens = 1.00**<br>**Spec = 1.00**	AUC = 0.93<br>Sens = 1.00<br>Spec = 0.85	**CysC**<br>(mg/l)
**AUC = 1.00**<br>**Sens = 1.00**<br>**Spec = 1.00**	**AUC = 1.00**<br>**Sens = 1.00**<br>**Spec = 1.00**	AUC = 0.93<br>Sens = 0.90<br>Spec = 0.92	**eGFR CysC**<br>(ml/min/1,73m^2)
AUC = 0.75<br>Sens = 0.57<br>Spec = 0.95	AUC = 0.65<br>Sens = 0.57<br>Spec = 0.90	AUC = 0.79<br>Sens = 1.00<br>Spec = 0.50	**uCR**<br>(mg/dl)
AUC = 0.74<br>Sens = 0.86<br>Spec = 0.59	AUC = 0.71<br>Sens = 0.77<br>Spec = 0.70	AUC = 0.55<br>Sens = 0.30<br>Spec = 0.92	**uProtein**<br>(mg/dl)
AUC = 0.89<br>Sens = 1.00<br>Spec = 0.82	AUC = 0.86<br>Sens = 1.00<br>Spec = 0.70	AUC = 0.68<br>Sens = 0.70<br>Spec = 0.67	**UPCR**<br>(mg/mg)
**AUC = 1.00**<br>**Sens = 1.00**	**AUC = 1.00**<br>**Sens = 1.00**	AUC = 0.52<br>Sens = 0.60	**sPOST**<br>(pg/ml)

**Spec = 1.00**	**Spec = 1.00**	Spec = 0.77	
**AUC = 1.00**<br>**Sens = 1.00**<br>**Spec = 1.00**	**AUC = 1.00**<br>**Sens = 1.00**<br>**Spec = 1.00**	AUC = 0.55<br>Sens = 1.00<br>Spec = 0.25	**uPOST**<br>(pg/ml)
AUC = 0.82<br>Sens = 0.57<br>Spec = 0.95	AUC = 0.73<br>Sens = 0.48<br>Spec = 1.00	AUC = 0.79<br>Sens = 0.88<br>Spec = 0.67	**uPOST /CR**<br>(pg/mg)
AUC = 0.89<br>Sens = 0.65<br>Spec = 1.00	AUC = 0.78<br>Sens = 0.65<br>Spec = 1.00	AUC = 0.94<br>Sens = 1.00<br>Spec = 0.83	**FePOST**<br>(%)
**AUC = 1.00**<br>**Sens = 1.00**<br>**Spec = 1.00**	**AUC = 1.00**<br>**Sens = 1.00**<br>**Spec = 1.00**	AUC = 0.62<br>Sens = 0.80<br>Spec = 0.62	**sKIM-1**<br>(pg/ml)
**AUC = 1.00**<br>**Sens = 1.00**<br>**Spec = 1.00**	**AUC = 1.00**<br>**Sens = 1.00**<br>**Spec = 1.00**	AUC = 0.69<br>Sens = 0.50<br>Spec = 0.92	**uKIM-1**<br>(pg/ml)
AUC = 0.89<br>Sens = 0.74<br>Spec = 0.95	AUC = 0.83<br>Sens = 0.74<br>Spec = 0.88	AUC = 0.78<br>Sens = 1.00<br>Spec = 0.50	**uKIM-1/CR**<br>(pg/mg)
AUC = 0.88<br>Sens = 0.65<br>Spec = 1.00	AUC = 0.77<br>Sens = 0.65<br>Spec = 1.00	AUC = 0.94<br>Sens = 1.00<br>Spec = 0.83	**FeKIM -1**<br>(%)
AUC = 0.91<br>Sens = 0.70<br>Spec = 0.95	AUC = 0.91<br>Sens = 0.70<br>Spec = 1.00	AUC = 0.49<br>Sens = 0.50<br>Spec = 0.67	**FePOST/**<br>**FeKIM-1**
		AUC = 0.55<br>Sens = 1.00<br>Spec = 0.27	**UACR**<br>(mg/g)
		AUC = 0.74<br>Sens = 0.80<br>Spec = 0.69	**uα1**<br>(mg/l)
		AUC = 0.79<br>Sens = 0.80<br>Spec = 0.77	**uβ2**<br>(mg/l)
		AUC = 0.89<br>Sens = 1.00<br>Spec = 0.75	**uα1/CR**<br>(mg/g)
		AUC = 0.87<br>Sens = 1.00<br>Spec = 0.75	**uβ2/CR**<br>(mg/g)

CRP - C-reactive protein; CR – creatinine; eGFR – estimated glomerular filtration rate based on creatinine; BUN – blood urea nitrogen; CysC – cystatin C; eGFR CysC estimated glomerular filtration rate based on cystatin C; u – urine; UPCR – urine protein/creatinine ratio; sPOST-periostin in serum; uPOST- periostin in urine; sKIM-1 - KIM-1 in serum; uKIM-1- KIM-1 in urine; uPOST/CR- urine periostin/creatinine ratio; uKIM-1/CR- urine KIM-1/creatinine ratio; FePOST- fractional excretion of periostin; FeKIM-1 - fractional excretion of KIM-1; FePOST/FeKIM-1 - FePOST/FeKIM-1 ratio; UACR – urine albumin/creatinine ratio, uα1 - alfa-1 mikroglobulin in urine; uβ2-beta-2 mikroglobulin in urine, uα1/CR– urine alfa-1 mikroglobulin/creatinine ratio, uβ2/CR – urine beta-2 mikroglobulin/creatinine ratio.
